# Supplementary material for: Anti-Inflammatory Effects of (9Z,11E)-13-Oxooctadeca-9,11-dienoic Acid (13-KODE) Derived from Salicornia herbacea L. on Lipopolysaccharide-Stimulated Murine Macrophage via NF-kB and MAPK Inhibition and Nrf2/HO-1 Signaling Activation
Source: Antioxidants (Basel). 2022 Jan 18;11(2):180. doi: 10.3390/antiox11020180 (PMC8868157; doi:10.3390/antiox11020180)
Supplement: Supplementary file 1 [file antioxidants-11-00180-s001.zip › antioxidants-1544702-supplementary.pdf]

Table S1. Specific Real-time RT-qPCR primer sequences containing *iNOS, IL-1β, TNF-α,* and *β-actin* genes

Genes	Primers
iNOS	· Forward: 5′-CGAGACGGATAGGCAGAGATTG-3′<br>· Reverse: 5′-CTCTTCAAGCACCTCCAGGAA-3′
IL-1β	· Forward: 5′-GGGCCTCAAAGGAAAGAATC-3′<br>· Reverse: 5′-TACCAGTTGGGGAACTCTGC-3′
TNF-α	· Forward: 5′-CAGGCGGTGCCTATGTCTC-3′<br>· Reverse: 5′-CGATCACCCCGAAGTTCAGTAG-3′
β-actin	· Forward: 5′-CCACAGCTGAGAGGAAATC-3′<br>· Reverse: 5′-AAGGAAGGCTGGAAAAGAGC-3′

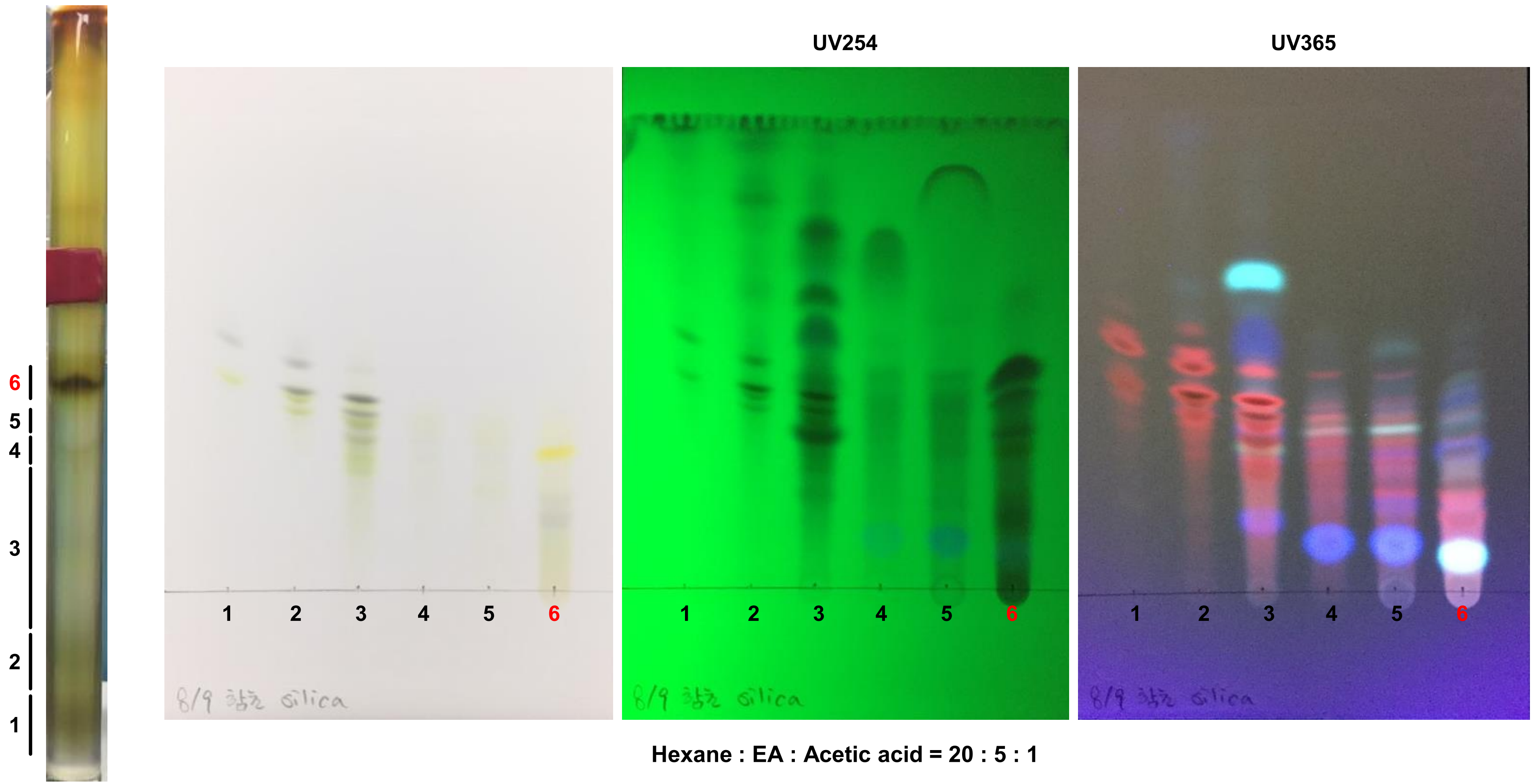

Supplementary Figure S1. Purification procedure of antioxidant from *Salicornia herbacea* L. extracts using SiO2 gel chromatography eluted with $CHCl_3$: MeOH (30:1). Active fraction: #6.

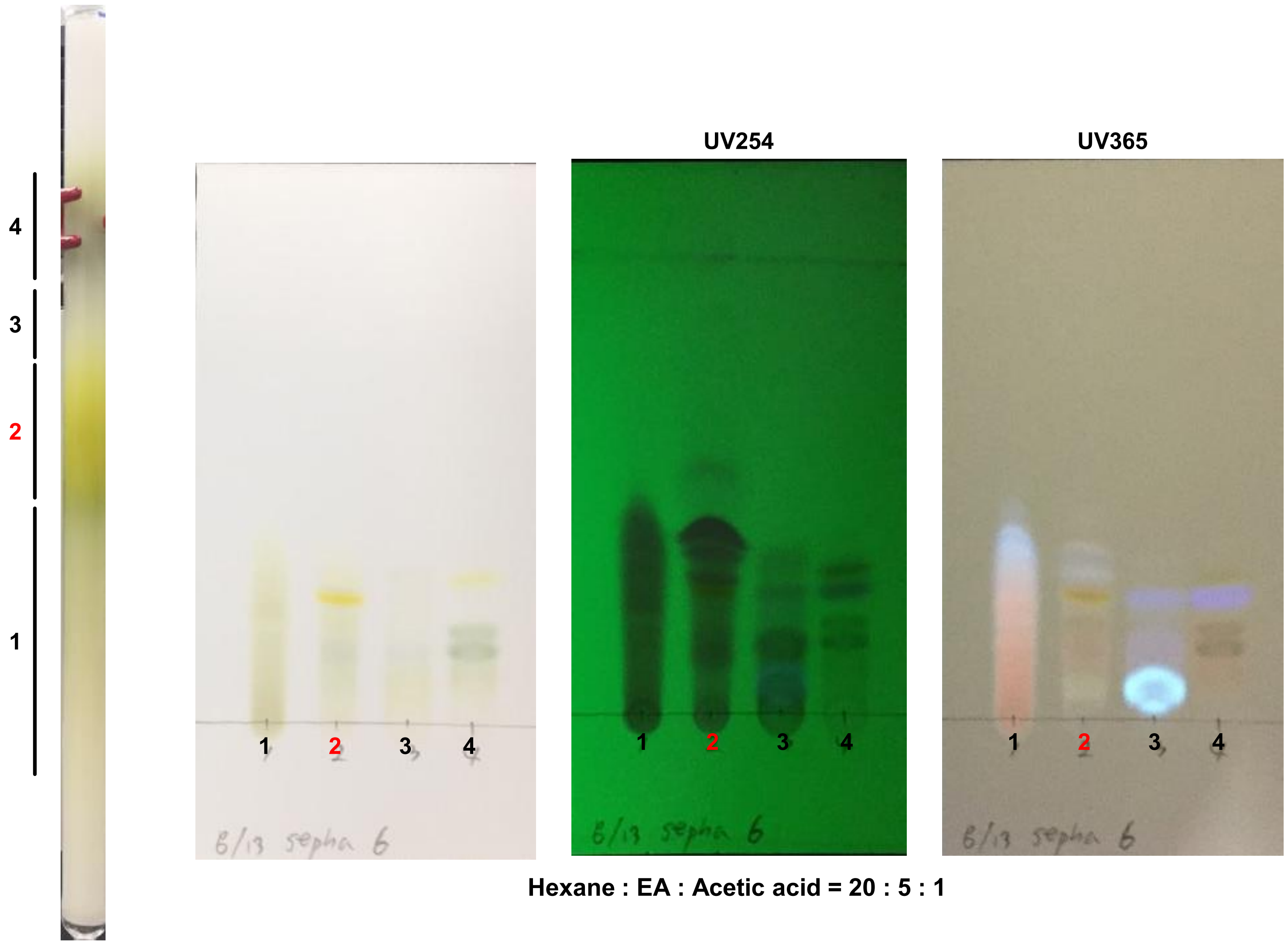

Supplementary Figure S2. Purification procedure of antioxidant from *Salicornia herbacea* L. extracts using sephadex LH-20 gel chromatography eluted with MeOH. Active fraction: #2.

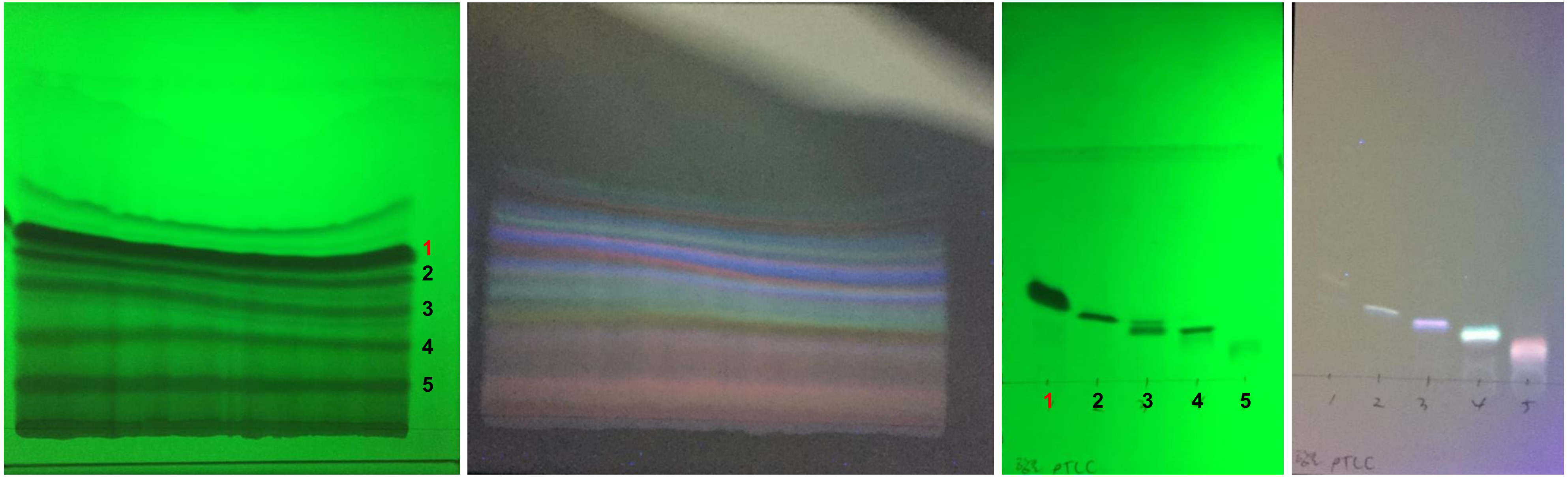

Hexane : EA : Acetic acid = 15 : 5 : 1 Hexane : EA : Acetic acid = 20 : 5 : 1

Supplementary Figure S3. Purification procedure of antioxidant from the *Salicornia herbacea* L. extracts using preparative thin layer chromatography with $CHCl_3$:MeOH (20:1). Active fraction: #1.

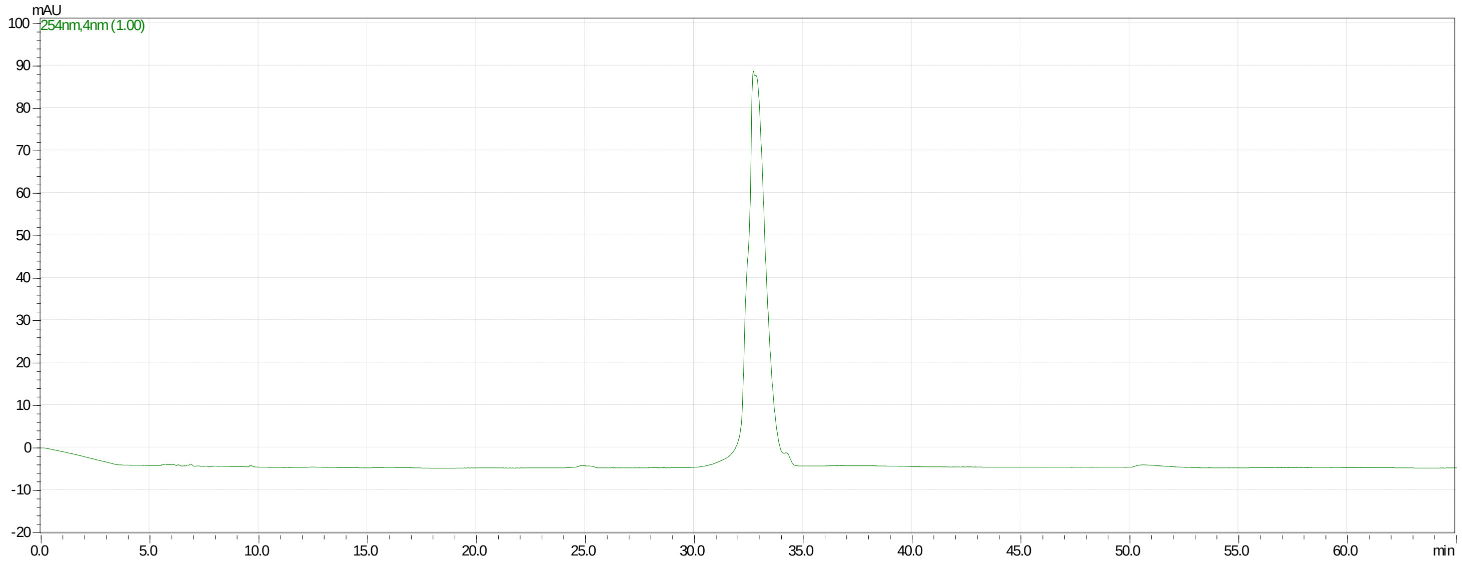

Supplementary Figure S4. Purification procedure of antioxidant from from the *Salicornia herbacea* L. extracts using Preparatory HPLC with acetonitrile.

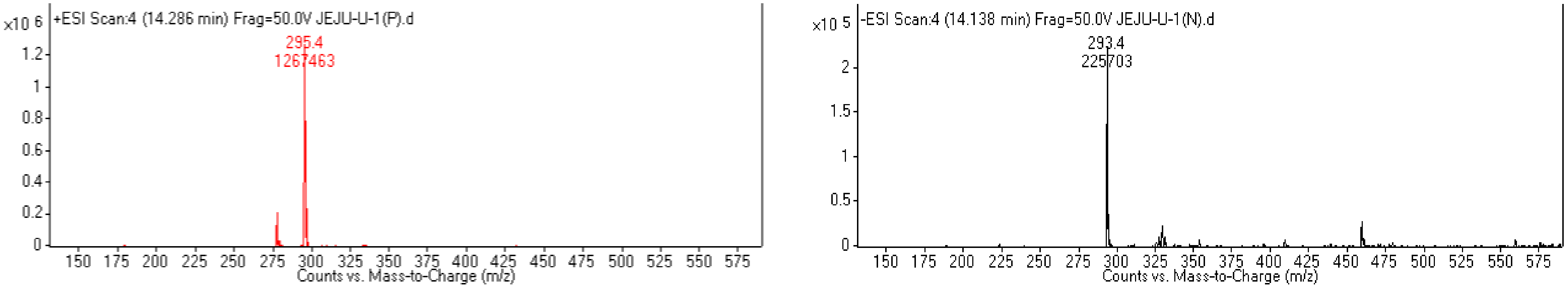

Supplementary Figure S5. ESI-mass spectrometry of the purified sample.

^{1}H-NMR

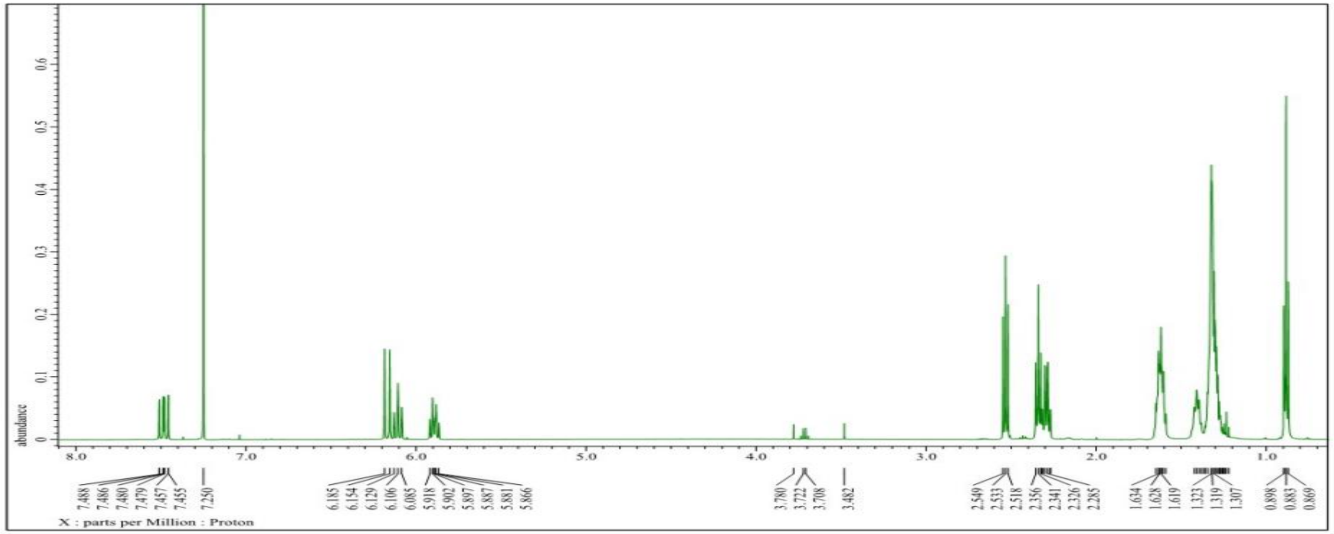

^{13}C-NMR

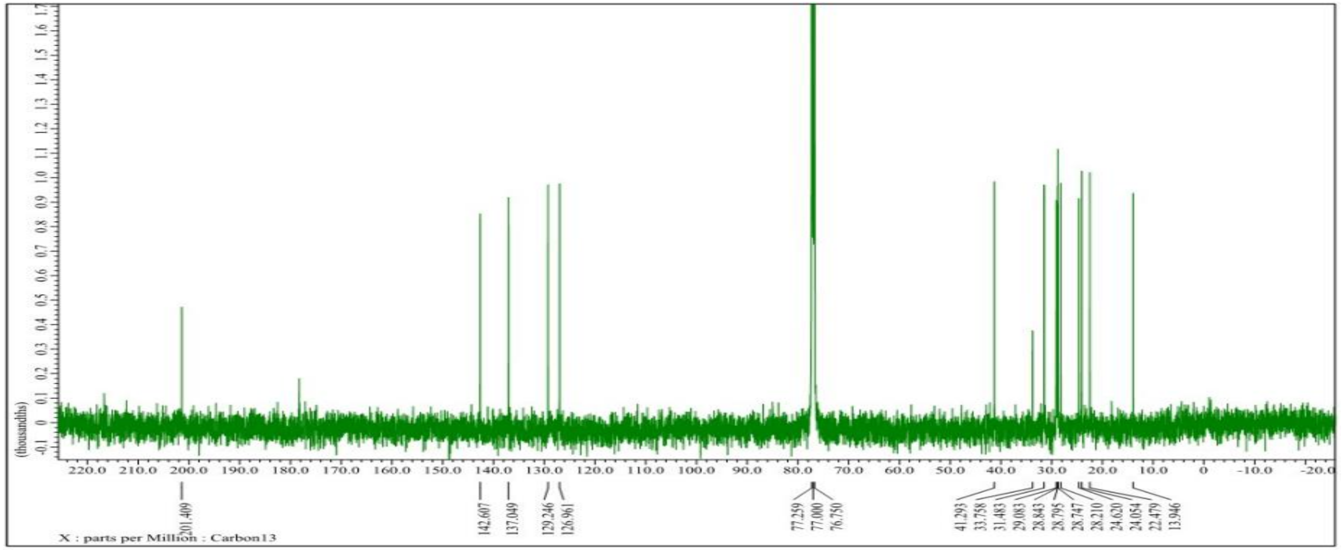

Supplementary Figure S6. ^{1}H NMR and ^{13}C NMR spectra of the purified sample.

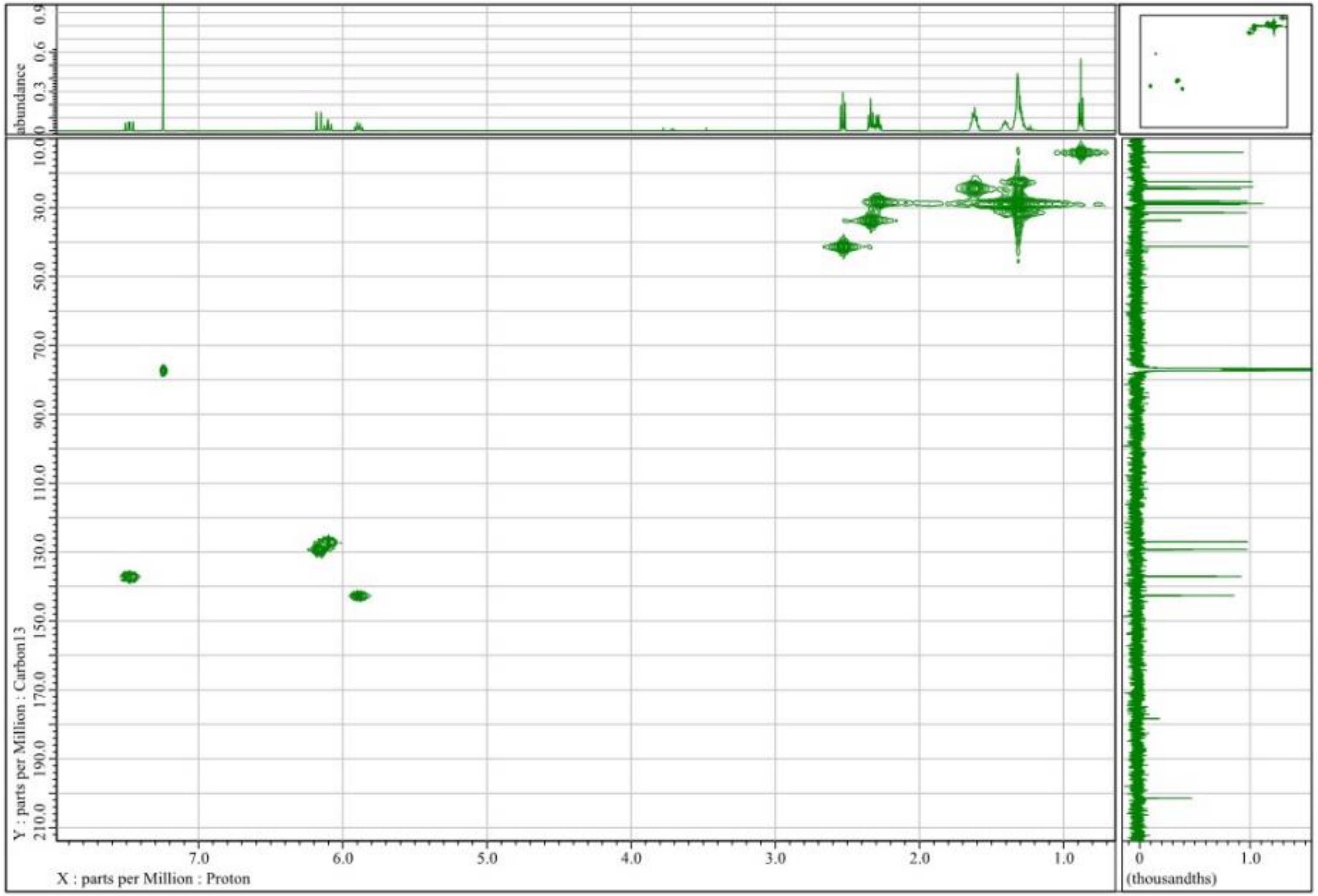

Supplementary Figure S7. **HMQC spectrum of the purified sample.**

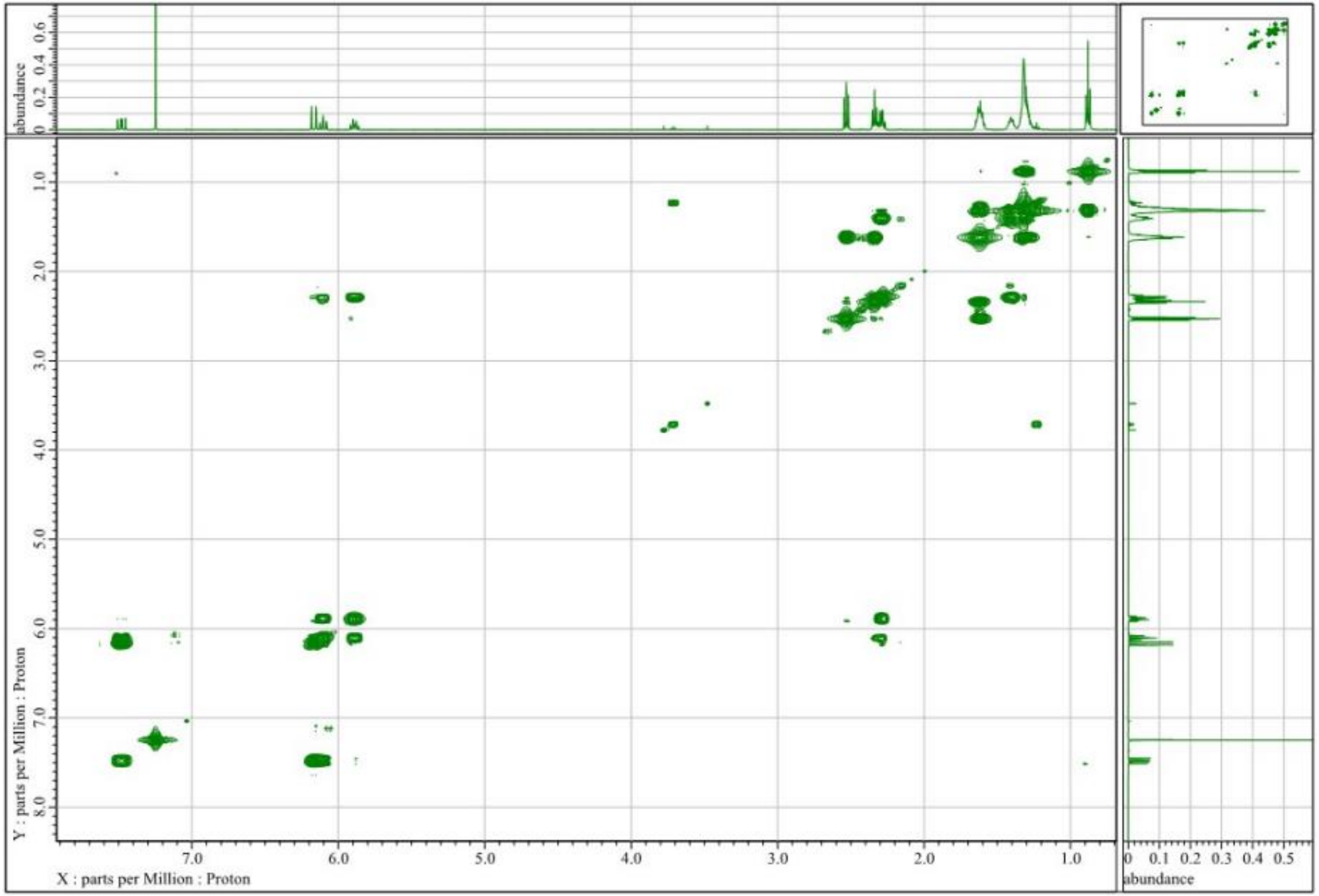

Supplementary Figure S8. ^{1}H-^{1}H COSY spectrum of the purified sample.

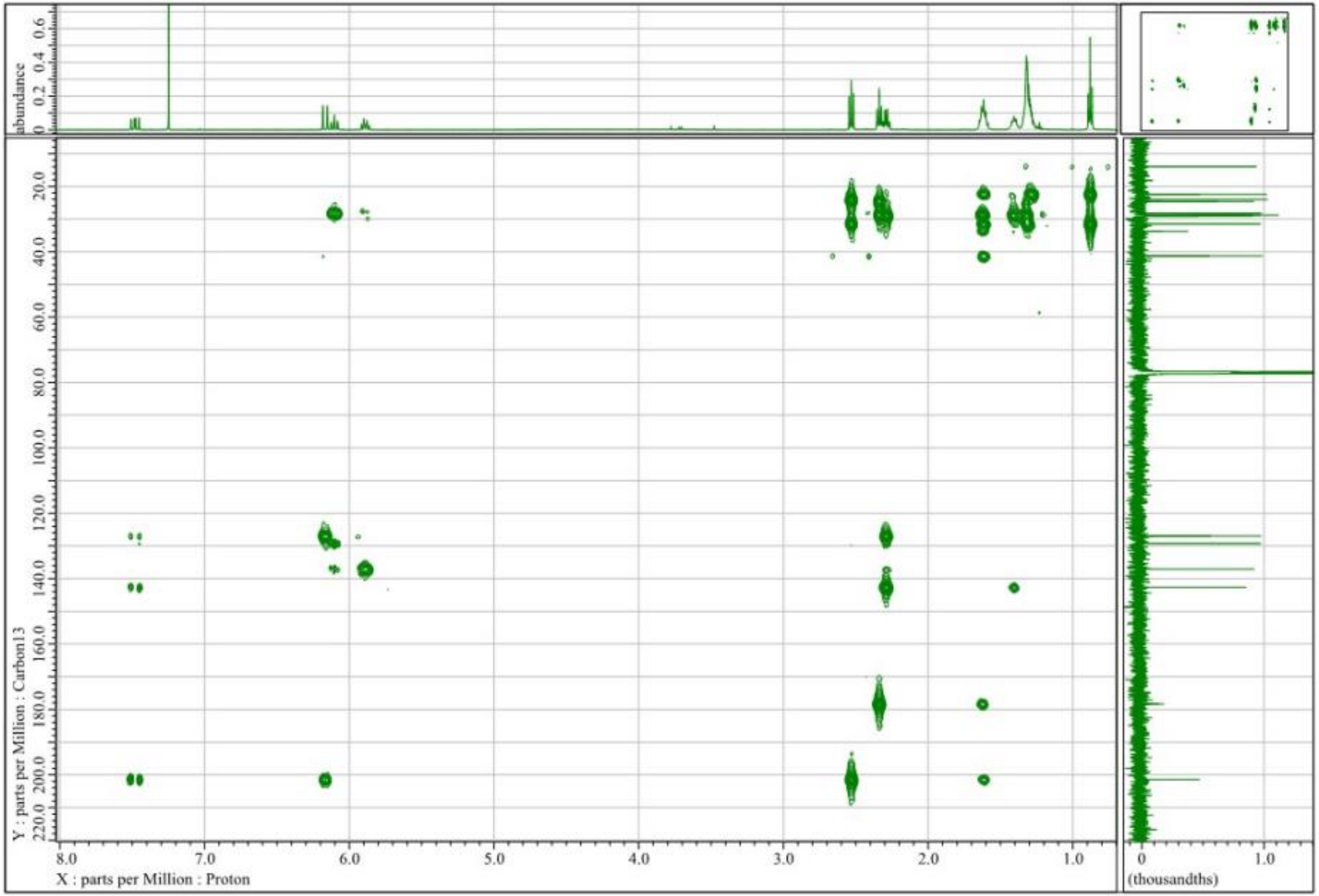

Supplementary Figure S9. HMBC spectrum of the purified sample.

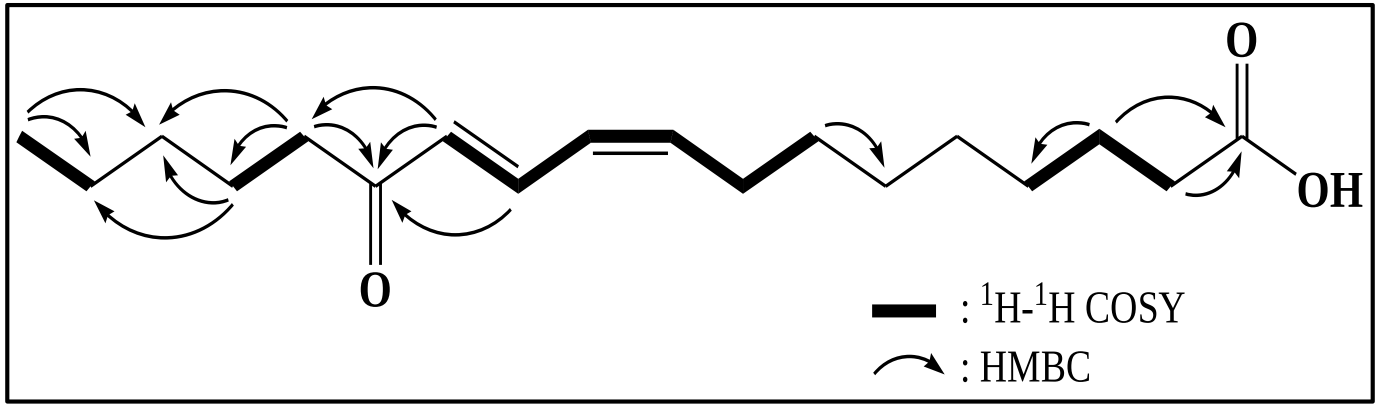

HMBC correlations

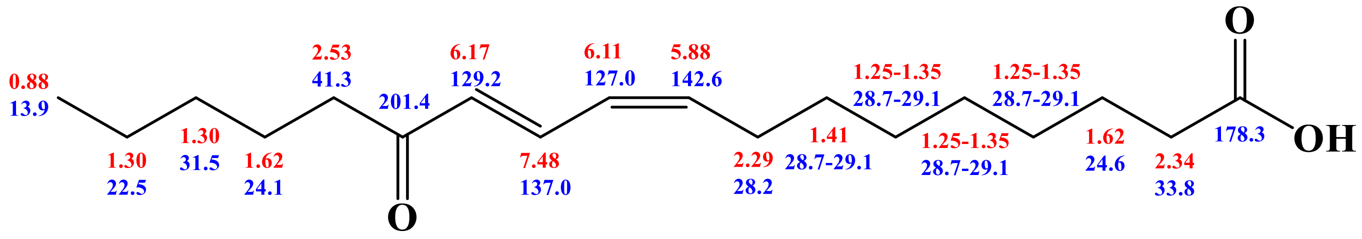

Supplementary Figure S10. **HMBC correlations and ^{1}H (red), ^{13}C (blue) peak assignments of the purified sample.**
